# Causes of an increased pressure gradient through the left ventricular outflow tract: a West Coast experience

**DOI:** 10.1007/s12574-017-0352-6

**Published:** 2017-09-18

**Authors:** Sayuki Kobayashi, Yoshihiko Sakai, Isao Taguchi, Hiroto Utsunomiya, Takahiro Shiota

**Affiliations:** 1 Center of Medical Ultrasonics, Koshigaya Hospital, Dokkyo Medical University, Saitama, Japan; 2 Department of Cardiology, Koshigaya Hospital, Dokkyo Medical University, 2-1-50 Minami Koshigaya, Koshigaya, Saitama 343-8555 Japan; 3 Department of Cardiovascular Medicine, Applied Life Sciences, Institute of Biomedical and Health Sciences, Hiroshima University, Hiroshima, Japan; 4 Cedars-Sinai Heart Institute, Los Angeles, CA USA

**Keywords:** Left ventricular outflow tract obstruction, Left ventricular outflow tract pressure gradient, Echocardiography, Negative inotropic agents

## Abstract

**Background:**

Left ventricular outflow tract obstruction (LVOTO) occurs from not only obstructive hypertrophic cardiomyopathy but also other conditions such as sigmoid septum or post mitral valve repair. However, the changes of the LVOT pressure gradient (LVOT PG) in LVOTO with various conditions remain unclear.

**Methods:**

The clinical characteristics and echocardiographic parameters of 73 patients with LVOT PG ≥50 mmHg at rest on Doppler ultrasound were retrospectively investigated.

**Results:**

In these patients (age 69 ± 15 years, 38% male), high prevalences of hypertension (66%) and anemia (43%) were observed. The most frequent clinical disease causing LVOTO was hypertrophic obstructive cardiomyopathy (HOCM) (74%). There were other conditions, including hypertensive left ventricular hypertrophy (9%), post-open heart surgery (7%), sigmoid septum (4%), hyperkinetic LV (3%), takotsubo cardiomyopathy (1.5%), and discrete subaortic membrane (1.5%). Significant improvement or reduction of LVOTO was observed in 93% of cases at follow-up (mean 44 months) echocardiography compared with the initial one with the use of medications and transcatheter procedures.

**Conclusions:**

The causes of LVOTO are diverse. However, the occurrence of LVOTO might depend on the coexistence of primary morphological LV characteristics and hemodynamic LV status. Specific factors causing LVOTO need to be investigated, and efforts for improvement of each individual status by the appropriate approach are required.

## Introduction

A left ventricular outflow tract pressure gradient (LVOT PG) ≥50 mmHg at rest in hypertrophic cardiomyopathy (HCM) is a predictor of heart failure and cardiovascular death [1, 2]. The clinical indication for myectomy and alcohol septal ablation is also LVOT PG ≥50 mmHg at rest or with physiological exercise [3]. We also encounter patients with LVOT obstruction (LVOTO) from other conditions such as sigmoid septum and post-surgical mitral valve repair or replacement. Case reports of such patients [4–6] exist. However, the changes of LVOT PG in patients with various causes remain unclear. Therefore, the clinical characteristics and clinical courses of patients with LVOT PG ≥50 mmHg at rest in Cedars-Sinai Heart Institute were investigated.

## Methods

### Study subjects

The present study was retrospectively designed. The study population consisted of 73 patients from the echocardiographic database of Cedars-Sinai Medical Center, Los Angeles, CA, with LVOT PG ≥50 mmHg at rest on Doppler ultrasound between January 2011 and February 2016. The study protocol was approved by the Cedars-Sinai Medical Center Institutional Review Board. Clinical data included age, sex, documented diagnosis of hypertension (HT), dyslipidemia (DL), diabetes mellitus (DM), anemia (hemoglobin level: male <14 mg/dl, female <12 mg/dl) and others are shown in Table 1.

**Table 1:** Baseline characteristics of patient with left ventricular outflow tract obstruction

Valuable	Value (*n* = 73)
Age (years)	69 ± 15
Male	28 (38%)
Follow-up periods (months)	45 ± 38
Hypertension	49 (66%)
Dyslipidemia	30 (41%)
Diabetes mellitus	10 (14%)
Coronary artery disease	23 (31%)
Chronic kidney disease	18 (24%)
Chronic lung disease	18 (24%)
Anemia	32 (43%)
Paroxysmal atrial fibrillation or paroxysmal supra ventricular tachycardia	22 (30%)
Cerebral infarction	3 (4%)
Paced lead	3 (4%)
Infective endocarditis	2 (3%)
Admission for heart failure	5 (7%)
Death	9 (12%)

### Transthoracic echocardiographic examinations

LVOT PG ≥50 mmHg at rest was defined as LVOTO, and it was compared between initial and follow-up transthoracic echocardiography (TTE). TTE was performed by two-dimensional (2D) Doppler echocardiography using a Philips iE33 ultrasound system (Philips Medical Systems, Andover, MA, USA) in clinically stable patients. LVOT PG was determined with quantitative Doppler using Bernoulli’s equation. The end-diastolic left ventricular diameter (LVDd), end-systolic LV diameter (LVDs), LV septal wall thickness, LV posterior wall thickness, and left atrial dimension (LAD) were measured in the parasternal long-axis view. Left atrial area was measured during LV end-systole using the biplane area-length method in the apical four-chamber view and indexed for body surface area. Left ventricular ejection fraction (LVEF) was calculated using the biplane method of discs (modified Simpson rule). To assess diastolic function, peak early diastolic velocity (*E*), peak late diastolic velocity (*A*), *E*/*A* ratio and deceleration time (DT) were derived from Doppler recordings of transmitral flow (TMF). Peak early diastolic mitral annular velocity (*e*′) of the lateral site of the mitral annulus was measured by tissue Doppler imaging. The ratio between *E* and *e*′ (*E*/*e*′) was calculated to estimate LV filling pressure. Systolic pulmonary artery pressure (SPAP) was estimated using the modified Bernoulli equation, measuring the peak systolic pressure gradient of the tricuspid valve regurgitant jet by continuous wave Doppler, and adding it to the estimated right atrial pressure. The severity of aortic valve regurgitation (AR), mitral valve regurgitation (MR), and tricuspid valve regurgitation (TR) was defined by the ACC/AHA task force on practice guidelines [7]. HCM was clinically diagnosed in the absence of HT or valve disease or other cardiomyopathies as restrictive cardiomyopathy, showing thickness of the LV septal wall (>15 mm) in one or more LV myocardial segments on M-mode or 2D echocardiography and asymmetrical septal hypertrophy (septal to LV posterior wall thickness ratio >1.3) [3]. However, patients with well-controlled HT were involved in the study. Systolic anterior motion of the mitral valve (SAM) was defined as severe when the anterior mitral leaflet or chordae contacted the septal wall of the LV during systole on 2D or M-mode echocardiography [8]. A hyperkinetic LV was defined as LVEF >0.70 without other abnormalities [9]. The echocardiographic data of the initial and follow-up TTE examinations were compared with mean follow-up periods of 44 ± 39 months. The clinical diagnoses causing LVOTO were classified as follows. Left ventricular hypertrophy (LVH) was defined in patients with a past history of long-term HT, without a diagnosis of HCM in the past, and with diffuse LV hypertrophy with LV wall thickness greater than 13 mm on TTE. A sigmoid septum [10] was as an angle between the LV septum and ascending aorta of less than 110°, but with wall thickness of the diastolic LV basal septum less than 15 mm.

### Statistical analysis

IBM SPSS version 19.0 (SPSS Inc, Chicago, IL, USA) was used for statistical analysis. Continuous variables are expressed as mean ± SD. Differences between continuous variables were assessed using Student’s *t* test for paired quantitative variables. Categorical variables are expressed as absolute values and frequency percentages and were compared using the *χ* ^2^ or Fisher’s exact test, as appropriate. Significance was defined by *p* < 0.05.

## Results

Table 1 lists the baseline characteristics of patients with LVOTO. For the total of 73 patients (age 69 ± 15 years, 38% male), high prevalences of hypertension (66%), anemia (43%), and PAF and PSVT (30%) were observed. Initial echocardiographic data of patients with LVOTO are listed in Table 2. SAM was recognized in most patients (91%) and severe SAM with septal contact of the anterior mitral leaflet or chordae, was recorded in more than half of patients (61%). Mitral annular calcification was observed in 39% of patients. Small LV and mildly dilated LA were found in more than 86% of the patients. More than moderate valve regurgitation was seen in 44% of patients.

**Table 2:** Initial echocardiographic data of patient with left ventricular outflow tract obstruction

Valuable	Value (*n* = 73)
Heart rate, beats per min	77 ± 18
Systolic anterior motion	67 (91%)
Severe systolic anterior motion	45 (61%)
Hyperkinetic LV (LV ejection fraction >0.70)	46 (62%)
Mitral annular calcification	29 (39%)
LV geometry	
End-diastolic diameter, mm	41 ± 6
End-systolic diameter, mm	24 ± 5
Septal wall thickness, mm	16 ± 4
Posterior wall thickness, mm	12 ± 3
LV systolic parameter	
Ejection fraction, %	74 ± 9
LV diastolic parameters	
E wave, cm/s	110 ± 43
A wave, cm/s	107 ± 43
Deceleration time, ms	229 ± 76
*E*/*e*′	15 ± 7
Systolic pulmonary arterial pressure, mmHg	34 ± 14
Left atrial parameters	
Left atrial diameter, mm	42 ± 9
Left atrial area index, cm^2^/m^2^	24 ± 6
Aortic valve regurgitation (none/mild/moderate/severe)	43/28/2/0
Mitral valve regurgitation (none/mild/moderate/severe)	3/38/22/10
Tricuspid valve regurgitation (none/mild/moderate/severe)	3/65/4/1

*LV* left ventricle, *E* peak early diastolic mitral flow velocity, *A* peak late diastolic mitral velocity, *E/e′* the ratio between E and peak early diastolic mitral annular velocity (e′) of the lateral site of the mitral annulus

### Cause of LVOTO

The most frequent clinical disease causing LVOTO was hypertrophic obstructive cardiomyopathy (74%) (Table 3). However, there were other conditions, including hypertensive LVH (9%), post-open heart surgery (mitral valve repair due to mitral regurgitation in four patients, both mitral and aortic valve replacement in one patient) (7%), sigmoid septum (4%), hyperkinetic LV (3%), takotsubo cardiomyopathy (1.5%), and discrete subaortic stenosis (1.5%). The clinical characteristics and therapy classified by clinical diagnosis are shown in Table 4. All patients with LVH (age 81 ± 14 years), sigmoid septum (age 85 ± 8 years) and takotsubo cardiomyopathy (age 88 years) were elderly. Anemia was found in patients with LVH (hemoglobin level 10.0 ± 1.8 mg/dl), hyperkinetic LV (hemoglobin level 10.9 ± 2.4 mg/dl), and takotsubo cardiomyopathy (hemoglobin level 9.5 mg/dl). Cardiac or sudden death occurred in three patients; one patient with hypertrophic obstructive cardiomyopathy (HOCM) died due to infective endocarditis (IE), and two patients with LVH were found dead at home.

**Table 3:** Clinical diagnosis of left ventricular outflow tract obstruction

Clinical diagnosis	*n* = 73
Hypertrophic obstructive cardiomyopathy	54 (74%)
Hypertensive left ventricular hypertrophy	7 (9%)
Post-cardiac surgery (4 of MV repair, 1 of MV replacement)	5 (7%)
Sigmoid septum	3 (4%)
Hyperkinetic heart	2 (3%)
Takotsubo cardiomyopathy	1 (1.5%)
Discrete subaortic stenosis	1 (1.5%)

*MV* mitral valve

**Table 4:** Clinical characteristics and therapy classified by clinical diagnosis with left ventricular outflow tract obstruction

	HOCM	LVH	Post surgery	Sigmoid septum	Hyperkinetic LV	Takotsubo cardiomyopathy	Sub-aortic membrane
n	54	7	5	3	2	1	1
Age, years	69 ± 13	81 ± 14	66 ± 18	85 ± 8	56 ± 1	88	31
Men	21	3	3	0	1	0	0
Hypertension	36	7	3	2	1	0	0
Dyslipidemia	21	4	2	2	0	0	0
DM	6	3	1	0	0	0	0
PAF, PSVT	18	3	0	1	0	0	0
Paced lead	2	0	1	0	0	0	0
IE or suspected	2	0	0	0	0	0	0
Hb, mg/dl	11.8 ± 2.1	10.0 ± 1.8	12.8 ± 2.1	12.5 ± 0.4	10.9 ± 2.4	9.5	11.9
Heart failure	4	1	0	0	0	0	0
Cardiac death or suspected	1	2	0	0	0	0	0
Medical	49	7	4	2	0	1	1
β-blocker	35	4	4	2	–	1	1
Disopyramide	13	0	0	0	–	0	0
Verapamil	6	0	0	0	–	0	0
Amiodalone	6	0	0	0	–	0	0
Diuretic	14	1	1	0	–	0	0
Antiplatelet	27	1	4	2	–	1	1
Anticoagulant	9	1	1	0	–	0	0
Ablation (+medication)	7 (6)	0	0	0	0	0	0
Surgery (+medication)	0	0	0	0	0	0	1 (1)

*HOCM* obstructive hypertrophic cardiomyopathy, *LVH* left ventricular hypertrophy, *LV* left ventricle, *DM* diabetes mellitus, *PAF* paroxysmal atrial fibrillation, *PSVT* paroxysmal supra ventricular tachycardia, *IE* infective endocarditis, *Hb* hemoglobin

### Treatment

Forty-nine patients with HOCM were treated with combinations of negative inotropic agents such as disopyramide or antiarrhythmic agents such as amiodarone, or antiplatelet agents with and without β-blockers (Table 4). Alcohol ablation was performed for seven symptomatic HOCM patients who were refractory to medical therapy. LVOTO patients without HOCM had medical therapy mainly with β-blockers. Only one patient, who was diagnosed with a subaortic membrane, received surgical treatment, resection of the subaortic membrane, and septal myectomy.

### Echocardiographic parameters classified by clinical diagnosis

Echocardiographic parameters classified by clinical diagnosis are shown in Table 5. Patients with LVH and sigmoid septum showed smaller LV size than other patients, and patients with HOCM, LVH, and hyperkinetic LV had larger LVEF. *E*/*e*′ and SPAP were the highest in patients with LVH.

**Table 5:** Echocardiographic parameters classified by clinical diagnosis with left ventricular outflow tract obstruction

	HOCM	LVH	Post surgery	Sigmoid septum	Hyperkinetic LV	Takotsubo cardiomyopathy	Sub-aortic membrane
*n*	54	7	5	3	2	1	1
HR (bpm)	76 ± 16	81 ± 13	66 ± 18	95 ± 18	56 ± 1	87	80
Severe SAM	33	3	1	1	2	1	0
MAC	25	4	0	0	0	0	0
LVDd (mm)	42 ± 6	37 ± 6	45 ± 5	34 ± 3	40 ± 13	47	43
LVDs (mm)	24 ± 5	21 ± 5	28 ± 3	24 ± 1	21 ± 1	33	28
IVS th (mm)	17 ± 4	14 ± 2	12 ± 1	11 ± 3	10 ± 2	12	11
PW th (mm)	12 ± 2	14 ± 3	11 ± 1	10 ± 3	9 ± 1	10	11
LVEF (%)	76 ± 8	79 ± 8	66 ± 11	66 ± 5	79 ± 7	57	65
TMF, E, (cm/s)	106 ± 43	118 ± 27	132 ± 82	98 ± 23	126 ± 4	115	118
TMF, A (cm/s)	101 ± 35	138 ± 18	88 ± 36	81 ± 10		171	82
TMF, DT (ms)	225 ± 70	248 ± 108	280 ± 77	134 ± 19		346	191
*E*/*e*′	15.0 ± 8	18.8 ± 9.1	14.3 ± 4.4	10.0 ± 4.0	10.0 ± 1.0	14.7	11.2
SPAP (mmHg)	33 ± 13	45 ± 21	30 ± 6	40 ± 17		32	45
LAD (mm)	43 ± 8	39 ± 9	42 ± 8	32 ± 7	42 ± 6	43	33
LA area, cm^2^	25 ± 6	21 ± 6	24 ± 5	15 ± 8	20 ± 4	30	13
Severity of AR (none/mild/moderate/severe)	34/19/1/0	4/3/0/0	1/4/0/0	1/2/0/0	2/0/0/0	1/0/0/0	0/0/1/0
Severity of MR (none/mild/moderate/severe)	3/33/14/4	0/2/1/4	0/0/4/1	0/1/2/0	0/1/1/0	0/0/0/1	0/1/0/0
Severity of TR (none/mild/moderate/severe)	3/48/3/0	0/6/0/1	0/5/0/0	0/2/1/0	0/2/0/0	0/1/0/0	0/1/0/0

*HOCM* obstructive hypertrophic cardiomyopathy, *LVH* left ventricular hypertrophy, *LV* left ventricle, *HR* heart rate, *bpm* beats per min, *SAM* systolic anterior movement, *MAC* mitral annular calcification, *LVDd* end-diastolic left ventricular diameter, *LVDs* end-systolic left ventricular diameter, *IVS th* interventricular septum wall thickness, *PW th* posterior wall thickness, *LVEF* left ventricular ejection fraction, *TMF* transmitral flow, *E* peak early diastolic mitral flow velocity, *A* peak late diastolic mitral velocity, *DT* deceleration time, *E/e′* ratio between *E* and peak early diastolic mitral annular velocity (*e*′) of the lateral site of the mitral annulus, *SPAP* systolic pulmonary artery pressure, *LAD* left atrial diameter, *LA* left atrium, *AR* aortic valve regurgitation, *MR* mitral valve regurgitation, *TR* tricuspid valve regurgitation

### Follow-up echocardiography

**Fig. 1:**
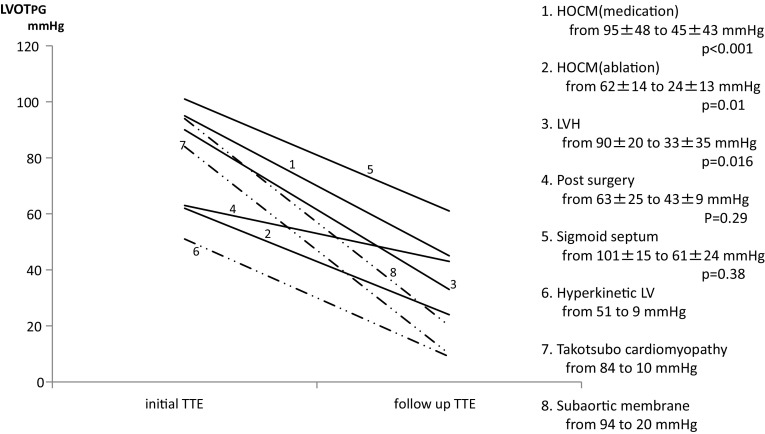
Comparison of left ventricular outflow tract peak pressure gradient classified by condition between initial and follow-up TTE examinations. *TTE* transthoracic echocardiography, *HOCM* hypertrophic obstructive cardiomyopathy, *LVH* left ventricular hypertrophy, *LV* left ventricle

**Fig. 2:**
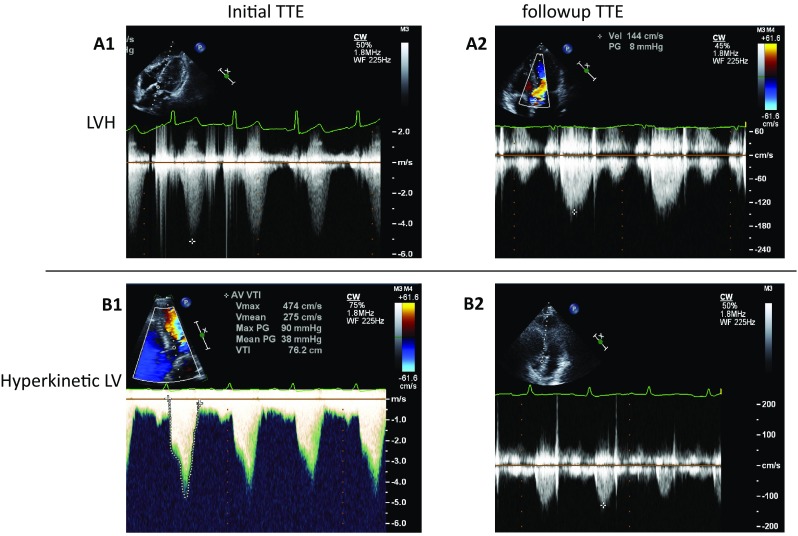
Examples of left ventricular outflow on continuous-wave Doppler echocardiography at initial and follow-up TTE in cases with LVH and hyperkinetic LV compared with LVOT PG (A1: 106 mmHg in a case with LVH, B1: 90 mmHg in a case with hyperkinetic LV) at the initial TTE, LVOT PG (A2: 8 mmHg in a case with LVH, B2: 7 mmHg in a case with hyperkinetic LV) at follow-up TTE showed decreases. *TTE* transthoracic echocardiography, *LVH* left ventricular hypertrophy, *LV* left ventricle, *LVOT PG* left ventricular outflow tract pressure gradient

Seventy patients (96%) were followed by TTE. Results are shown with comparisons of initial and follow-up TTE results (Fig. 1). LVOT PG decreased significantly from 95 ± 48 to 45 ± 43 mmHg (*p* < 0.001) in HOCM with drug therapy, from 62 ± 14 to 24 ± 13 mmHg (*p* = 0.01) in HOCM with alcohol septal ablation and from 90 ± 20 to 33 ± 35 mmHg (*p* = 0.016) in LVH compared with initial TTE. However, in post-surgery patients, there was no significant reduction of LVOT PG. Changes of LVOT PG could not be analyzed statistically in patients with hyperkinetic LV, takotsubo cardiomyopathy and subaortic membrane because of the small number of cases, but LVOT PG decreased in all of them. Examples of LV outflow by continuous wave Doppler echocardiography at initial TTE and follow-up TTE are displayed in Fig. 2. In both cases with LVH and hyperkinetic LV, LVOT PG decreased at follow-up TTE, compared with initial TTE (LVH from 106 to 8 mmHg, hyperkinetic LV from 90 to 7 mmHg).

## Discussion

The most frequent cause of LVOTO has been well known to be HOCM. Maron et al. [2] reported that an LVOT PG was present in 70% of patients with HCM at rest or with physiological exercise. In the present study, 74% of the LVOTO study population had HOCM. Most patients with HOCM were treated with negative inotropic agents such as β-blockers, and the LVOT gradient decreased in most of them. However, patients with HOCM with medically refractory symptoms such as shortness of breath, palpitation, and syncope, were successfully treated with alcohol septal ablation (ASA) in our heart institute.

Among other etiologies causing LVOTO, LVH was most frequently observed (9% of the study population) in the present series. In this group, multiple clinical characteristics including old age (mean age 81 ± 14 years), hypertension (100%), anemia (mean hemoglobin level 10.0 ± 1.8 mg/dl), small LV cavity (mean LVDd 37 ± 6 mm), hyperkinetic LV function (mean LVEF 79 ± 8%) and high *E*/*e*′ (mean 18.8 ± 9.1) were observed. It was reported that LVOTO occurs if LV systolic function becomes hyperdynamic due to other factors in a patient with basal septal hypertrophy, and this tends to happen in elderly patients who have a history of HT [11]. Elderly persons often have HT, and basal septal hypertrophy or concentric LVH is often found. Therefore one or more factors causing hyperdynamic LV such as anemia, in addition to primary morphological LV characteristics, may easily result in LVOTO. Sgreccia et al. [12] reported that LVOTO was also observed in hypertensive patients with the diffuse type of LVH, normal cavity size, and normal or supernormal systolic LV function. These patients had a mean LVOT PG 83 ± 31 mmHg, and the gradients improved rapidly after changing from nitrates, digoxin, and diuretics to calcium channel antagonists or β-blockers. In the present study, β-blockers were used in four patients and angiotensin receptor blockers were used in two patients. LVOT PG decreased from 90 ± 20 mmHg at initial TTE to 33 ± 35 mmHg at follow-up TTE. In addition to the use of negative inotropic agents, it may be important to improve some other factors causing LVOTO, such as anemia and hypertension, and continue to follow echo parameters including LVOT PG. Even when four patients (80%) with MV repair were treated with β-blockers, only small reductions of LVOT PG were achieved at follow-up TTE. Geometric factors in which the anterior mitral coaptation line is displaced by surgical repair would cause LVOTO mainly in patients with MV repair [13]. Patients after surgery have rigid structural abnormalities with narrowing of the LVOT, rather than the hyperkinetic LV seen in HOCM. Therefore, negative inotropic agents such as β-blockers might decrease LVOT PG less in patients after surgery. On the other hand, Brown et al. [14] reported that most cases of SAM resolved with conservative measures including β-blockers after surgical mitral valve repair and late reoperation was not required. All of the present cases with surgical MV repair did not need reoperation and did not suffer any complications. Three patients with sigmoid septum also showed LVOTO, were elderly (mean age 85 ± 8 years), and had a small LV cavity (mean LVDd 34 ± 3 mm). The thickness of the IVS in the basal portion was 13 mm in two of three patients and 8 mm in one patient. The LVOT gradient improved from 101 ± 15 to 61 ± 24 mmHg on the follow-up TTE with use of β-blockers (67%). Ranasinghe et al. [15] reported that 15 patients with LVOTO due to sigmoid septum (*n* = 9) and LVH (*n* = 6) showed an LVOT PG of 74.4 ± 55.2 mmHg. A reduction in LVOT PG of 40.9 mmHg on β-blockers, with a further reduction of 24.2 ± 25.8 mmHg with added disopyramide, was observed. Other investigators [4] reported reduction of LVOT PG from 154 to 36 mmHg and from 56 to 16 mmHg in two cases with sigmoid septum after the use of cibenzoline (200 mg/day). These results may suggest that negative inotropic agents are effective for reduction of an LVOT PG due to sigmoid septum. Patients with hyperkinetic LV showed LVOT PG reduction after treatment for dehydration. A patient with takotsubo cardiomyopathy causing LVOTO in the present study showed advanced age and SAM-induced severe MR at the initial TTE. At the follow-up TTE, SAM disappeared, and the LVOT PG also improved rapidly with the natural course of the disease on β-blocker therapy. A recent study reported that up to 20% of takotsubo cardiomyopathy cases may develop LVOTO [16]. The mechanism of takotsubo cardiomyopathy remains unknown, but catecholamine-induced cardiotoxicity is generally a well-accepted therapy [17]. Therefore, it is reasonable to treat such patients with β-blockers.

From the above, LVOTO is diverse. LVOTO could occur when two or more structural LV characteristics and hemodynamic status were present together. The degree of involvement of these factors may vary by the disease and/or its status and LVOT PG may also show different changes by pharmacological or surgical interventions.

## Study limitations

In the present study, 43% of patients had anemia at the time of the initial TTE. It has been reported that moderate-to-severe anemia increases stroke volume and heart rate [18] and anemia activates the sympathetic nervous system [19]. Anemia could have contributed to the development of LVOTO at the time of initial TTE in the present study. Although an increased hemoglobin concentration was expected at the time of follow-up TTE, hemoglobin concentrations were not available at that time point. Therefore, whether anemia increased the LVOT PG could not be evaluated in the present study.

## Conclusions

The present results showed that LVOTO might depend on the coexistence of primary morphological LV characteristics and hemodynamic LV status. To improve LVOTO, we should investigate clinically relevant factors and manage the LVOTO accordingly.
